# The Effect of Fentanyl Abuse on the Gut Microbiota Pattern, Inflammation, and Metabolic Alterations in a Fentanyl Dependance Rat Model

**DOI:** 10.1155/mi/6661864

**Published:** 2025-08-03

**Authors:** Kianoosh Ferdosnejad, Parvaneh Maghami, Mohammad-Reza Zarrindast, Seyed Davar Siadat

**Affiliations:** ^1^Department of Biology, SR.C., Islamic Azad University, Tehran, Iran; ^2^Department of Pharmacology, School of Medicine, Tehran University of Medical Sciences, Tehran, Iran; ^3^Iranian National Center for Addiction Studies, Tehran University of Medical Sciences, Tehran, Iran; ^4^Institute for Cognitive Science Studies (ICSS), Tehran, Iran; ^5^Microbiology Research Center (MRC), Pasteur Institute of Iran, Tehran, Iran; ^6^Department of Mycobacteriology and Pulmonary Research, Pasteur Institute of Iran, Tehran, Iran

**Keywords:** dysbiosis, fentanyl, inflammation, metabolite, microbiota, synthetic opioid

## Abstract

**Introduction:** This study examines the effects of fentanyl misuse on gut microbiota, inflammation, and metabolic pathways using a rat model. As the opioid crisis, driven by synthetic opioids like fentanyl, escalates, identifying new biomarkers, and therapeutic strategies becomes crucial to mitigate its negative impacts and rising overdose cases.

**Methods:** This study was done using species-specific 16S rRNA gene profiling through absolute real-time PCR techniques, alongside relative real-time PCR analysis for inflammatory and metabolic markers and liquid chromatography–mass spectrometry (LC–MS) for metabolite quantification. To visualize the correlations between microbial abundance and inflammatory/metabolic markers, Spearman/Pearson correlation analyses were performed.

**Results:** Fentanyl treatment increased *Clostridium sensu stricto* (*p*=0.69) and *Bacteroides fragilis* (*p*=0.04), while *Faecalibacterium prausnitzii* (*p*=0.14) and *Lacticaseibacillus rhamnosus* (*p*=0.14) showed a tendency to decrease. Additionally, fentanyl-treated rats exhibited heightened levels of pro-inflammatory cytokines (*IL-1β*, *p*=0.01; *TNF-α*, *p*=0.083; *IL-6*, *p*=0.17) and increased expression of toll-like receptors (*TLR-2*, *p*=0.005; *TLR-4*, *p*=0.001), indicating intestinal inflammation. Metabolic pathway analysis revealed significant alterations, including increased *BCoAT* expression (*p*  < 0.001) and decreased *tph1* expression (*p*  < 0.001) in fentanyl-treated rats. LC–MS analysis indicated a significant reduction in butyrate levels (45.49 ± 7.82; 73.52 ± 5.4 µM; *p*  < 0.001), suggesting impaired short-chain fatty acid production and potential gut barrier integrity issues. Tryptophan levels were also significantly lower (14.96 ± 1.3; 22.38 ± 2.1 µM; *p*  < 0.001), indicating possible disruptions in serotonin synthesis, while deoxycholic acid levels increased (106.1 ± 7.3; 77.35 ± 3.5 µM; *p*  < 0.001), suggesting altered bile acid metabolism contributing to gut inflammation. Leucine levels (31.8 ± 1.5; 30.67 ± 1.6 µM; *p*=0.15) remained comparable between groups.

**Conclusion:** This study reveals complex relationships between fentanyl consumption, gut microbiota alterations, gut inflammation, and metabolic functions. The identified changes in specific bacterial species and inflammatory markers suggest a potential mechanism by which fentanyl may exacerbate gut inflammation and disrupt metabolic pathways, highlighting the importance of these dynamics in understanding the microbiota importance and opioid dependance.

## 1. Introduction

Substance addiction is a major preventable cause of death worldwide, with the opioid crisis being particularly challenging [1]. Fentanyl, a synthetic opioid, is especially concerning due to its potency, which is 50–100 times greater than morphine [2]. Since 2016, fentanyl has been responsible for a significant number of synthetic opioid overdoses, emphasizing its critical role in the crisis [1]. While it is an important analgesic for severe pain management [3], its misuse has led to a sharp increase in opioid-related fatalities [4, 5]. Understanding the mechanisms of fentanyl addiction is essential [6], as research indicates its high potential for habit formation, resulting in rapid physical and psychological dependance. Symptoms of dependency include intense cravings and a loss of control over use, often leading to continued consumption despite adverse effects [7, 8]. This situation highlights the urgent need for effective interventions [9].

Opioids exert their effects by binding to specific receptors in the body, particularly in the enteric nervous system (ENS) [10]. Key receptors involved include the delta opioid receptor (DOP), nociceptin/orphanin receptor (NOP), kappa opioid receptor (KOP), and mu opioid receptor (MOP) [11]. Fentanyl interacts with these receptors, affecting various cell types, including those in the ENS and immune system, which can disrupt digestive and immune functions [12, 13]. Recent findings underscore the role of gut microbiota in health and disease prevention [13]. Dysbiosis, or changes in microbial composition, has been linked to numerous health issues, with gut microbiota playing a crucial role in maintaining digestive barrier integrity, metabolizing dietary energy, preventing pathogen colonization, and modulating immune responses [14]. Notably, there is emerging evidence of a bidirectional relationship between gut microbiota and opioid addiction, mediated by immune mechanisms [15].

Fentanyl's interactions with immune and metabolic pathways can affect mucosal thickness, intestinal motility, and epithelial integrity [16]. Studies have shown associations between opioid abuse and gastrointestinal diseases, with evidence suggesting that opioid-induced dysbiosis contributes to gastrointestinal dysfunction [17, 18]. Chronic opioid exposure disrupts gut microbial balance, leading to increased intestinal permeability and systemic inflammation, which are hallmarks of many gastrointestinal disorders [13, 19]. This study investigates how fentanyl abuse disrupts gut microbiota composition and metabolic function, with particular focus on key bacterial species, including *Faecalibacterium prausnitzii*, *Lacticaseibacillus rhamnosus*, *Bacteroides fragilis*, and *Clostridium sensu stricto* due to their reciprocal interactions with opioid exposure. Specifically, we examine *F. prausnitzii* for its role in producing short-chain fatty acids (SCFAs) like butyrate that regulate gut inflammation, *L. rhamnosus* for its conversion of tryptophan to serotonin (impacting mood and pain perception relevant to addiction), *B. fragilis* for its modulation of bile acid metabolism and inflammatory responses, and *C. sensu stricto* for its influence on gut barrier function through amino acid fermentation. Additionally, the study explores the relationship between fentanyl-induced microbiota alterations and changes in inflammatory gene expression, aiming to uncover mechanistic insights into how gut dysbiosis may exacerbate opioid addiction and related gastrointestinal dysfunction.

## 2. Materials and Methods

### 2.1. Experimental Animals

Twenty adult male Wistar rats, weighing between 220 and 250 g, were obtained from the Pasteur Institute of Iran and placed in Plexiglas enclosures that can accommodate a maximum of four rats per enclosure. The rats were provided with unlimited access to both tap water and food, while the enclosures preserved a 12 h light-dark cycle, with a temperature set at 22 ± 2°C and humidity levels at 55% ± 5% [20]. The research procedures were sanctioned by the Research and Ethics Committee of the School of Medicine, Tehran University of Medical Sciences (IR TUMS.AEC.1402.115), and were performed in compliance with the guidelines outlined in the National Institutes of Health Guide for the Care and Use of Laboratory Animals.

### 2.2. Animal Treatment

The experiments were conducted with Fentanyl obtained from Tofigh Daru Research & Engineering Company in Tehran, Iran. Following a 1 week acclimatization period, the rats were randomly allocated into two groups;one group was subjected to fentanyl treatment and the other was designated as controls, with each group comprising 10 rats. Each experimental condition was replicated two times, resulting in a total of 20 rats per group. The experimental design was developed to evaluate the impact of fentanyl administration on the microbiota composition and inflammatory responses in the rats. Fentanyl was dissolved in sterile saline and delivered through intraperitoneally (IP) injections to the treatment groups for a consecutive 10-day period. The dosing schedule required gradual increases of 5, 10, 15, 20, and 25 micrograms per kilogram of body weight per day. The control cohorts were administered equivalent quantities of saline via IP injections once daily over the same duration [21].

### 2.3. Fecal Sample Collection and DNA Extraction

Stool samples were transferred in 1.7 mL RNase/DNase-free tubes (Catalog #: C-2170, Denville Scientific, Holliston, MA, USA) for DNA extraction. These samples were immediately frozen on ice and then stored at −80°C until further processing. DNA extraction from the fecal was carried out using the QIAamp DNA Mini Kit from Qiagen (Qiagen, Valencia, CA, USA). Following the manufacturer's instructions, 0.2 g of fecal samples were utilized for DNA extraction using QIAamp DNA Mini Kits. Gel electrophoresis, along with a Nanodrop spectrophotometer (ND-1000; Nanodrop Technologies), was employed to assess the quality and concentration of the extracted DNA. All DNA samples obtained were stored at −80°C until amplification [22].

### 2.4. Quantitative Real-Time PCR Amplification for Detection of Specific Genera

Quantitative real-time PCR was employed to quantify the absolute genomic abundances of various bacterial species in fecal samples. The selection of bacterial targets, including *L. rhamnosus*, *F. prausnitzii*, *B. fragilis*, and *C. sensu stricto*, was based on their known roles in gut health and disease, as documented in microbiota databases like Disbiome (https://disbiome.ugent.be/) [23] and supported by previous literature [24–26]. This targeted approach was adopted due to technical constraints that limited the feasibility of broader methods, such as 16S rRNA sequencing or metagenomic analyses. By focusing on specific bacterial species, we aimed to gain insights into their relative abundances and potential functional roles within the gut microbiota, which aligns with the study's conceptual novelty in addressing its research question.

The amplification of specific primers was standardized against the 16SrRNA gene, and primer specificity was confirmed through nucleotide BLAST analysis on NCBI. Details of the universal primers for the 16SrRNA gene can be found in Table 1. PCR reactions were completed in duplicate using the Roche LightCycler 96 system (Roche, Switzerland), with each 20 μL reaction mixture consisting of 8 μL distilled water, 10 μL syber green master mix (Takara, Japan), 0.5 μL forward and reverse primers, and 1 μL DNA template. The amplification process complicated 40 cycles of denaturation, annealing, and extension. The denaturation step at 95°C for 15 s, followed by annealing at 58°C for 20 s, and extension at 72°C for 20 s. A melting curve analysis was performed by cooling the product from 95° to 60°C, following amplification cycles. Microbial identification was performed at the species level, allowing for a focused analysis of the selected bacterial targets. Bacterial loads were determined by preparing 10-fold serial dilutions of DNA extracts from *Escherichia coli* standard strains and calculating DNA concentrations for each bacterium from fecal samples using the standard curve [27].

### 2.5. Detection of Inflammatory Cytokines by Real-Time PCR Analyses

To detect messenger RNA levels of inflammatory cytokines (*IL-1β*, *TNF-α*, and *IL-6*) and toll-like receptors (*TLR-2* and *TLR-4*) in the intestines, the anesthetized rats were decapitated and intestinal tissue were meticulously collected and immediately frozen in liquid nitrogen before being archived at −80°C until analysis. Tissues were homogenized by adding normal saline and total RNA extraction from homogenized intestinal specimens was carried out using TRIzol (Thermo Fisher Scientific, Inc.) reagent. A reverse transcription reaction was then performed to generate complementary DNA (cDNA) utilizing total RNA, an oligo (dT) primer, and ReverTra Ace (Toyobo, Osaka, Japan) as a reverse transcriptase. Subsequently, RT-PCR analyses were conducted to assess the expression levels of *IL-1β*, *TNF-α*, *IL-6*, *TLR-2*, and *TLR-4* in rat intestine using the obtained cDNA [28]. Each reaction was performed in duplicate, ensuring consistency in results. Primers for the PCR were designed with Oligo7 Primer Analysis Software version 7.60 (Molecular Biology Insights, Inc., Colorado Springs, CO) and are provided in Table 2.

### 2.6. Detection of Metabolic Pathways by Real-Time PCR Analyses

This study aims to investigate the differences in specific metabolic pathways between fentanyl-addicted and healthy control rats using quantitative real-time PCR to analyze gene expression related to specific metabolic functions. The selection of metabolic pathways was based on the roles of key gut bacteria in opioid-associated dysbiosis. Briefly, *F. prausnitzii* produces anti-inflammatory SCFAs, particularly butyrate, which supports gut barrier integrity. *L. rhamnosus* converts tryptophan to serotonin, influencing mood regulation, and pain perception—key factors in addiction. *B. fragilis* modulates bile acid metabolism and inflammatory responses, while *C. sensu stricto* contributes to gut barrier function through amino acid fermentation.

After the treatment period, homogenized intestinal tissues were collected from both groups of rats. Total RNA was extracted from the collected intestinal tissues using TRIzol (Thermo Fisher Scientific, Inc.) reagent. The extracted RNA was converted into cDNA via reverse transcription. Specific primers targeting genes associated with metabolic pathways of interest (including butyryl-CoA:acetate CoA-transferase (*BCoAT*) for SCFA production, tryptophan hydroxylase (*tph1*) for serotonin synthesis, 7 α-dehydroxylase (*baiCD*) for bile acid metabolism, and branched chain amino acid (*BCAA*) for amino acid fermentation) were designed and provided in Table 2. Quantitative PCR (qPCR) was performed in duplicates to measure the expression levels of these target genes in both fentanyl-addicted and healthy control rats. Relative gene expression was normalized to the housekeeping gene *gapdh*, and statistical analyses were conducted to compare pathway activity between groups.

### 2.7. Detection of Metabolic Pathways by Liquid Chromatography–Mass Spectrometry (LC–MS) Analyses

To assess levels of butyrate (as a SCFAs metabolite), tryptophan (as serotonin metabolite), deoxycholic acid (as bile acid metabolite), and leucine (as BCAA metabolite) in the intestines of fentanyl-dependent rats versus control groups, we initiated sample collection from the fentanyl dependance rat model. The selected metabolites were chosen based on the roles of specific selected gut bacteria (*L. rhamnosus*, *F. prausnitzii*, *B. fragilis*, and *C. sensu stricto*), as well as the selected metabolic genes (*BCoAT*, *tph1*, *baiCD*, and *BCAA*). Approximately 50 mg of the intestinal tissue was homogenized in 1 mL of cold extraction solvent, consisting of 80% methanol in water, using a tissue grinder to obtain a uniform suspension. This homogenate was then subjected to centrifugation at 12,000 × *g* for 10 min at 4°C, allowing for the separation of the supernatant which was retained for further analysis. Precipitation was performed using cold acetonitrile, followed by centrifugation to remove insoluble debris. The resulting supernatant was dried under nitrogen gas and reconstituted in a mobile phase-compatible solvent (0.1% formic acid in water/acetonitrile) prior to LC–MS analysis.

For LC–MS analysis, a reverse-phase column C18 (2.1 × 100 mm, 1.7 μm particle size) was employed to effectively separate the metabolites. A binary mobile phase was established, comprised of solvent A (water with 0.1% formic acid) and solvent B (acetonitrile with 0.1% formic acid), with a typical flow rate set between 0.2–0.5 mL/min. A volume of 10–20 µL of the filtered sample extract was injected into the LC system. Mass spectrometry was conducted using both positive and negative ion modes, aligning source parameters according to the manufacturer's specifications. Multiple reaction monitoring (MRM) settings were established for quantification of butyrate, tryptophan, deoxycholic acid, and leucine, alongside their respective internal standards, ensuring precise detection and measurement. The data acquisition involved generating calibration curves utilizing known concentrations of the target metabolites. Subsequent analysis of the LC–MS data was performed using dedicated software to facilitate peak identification and quantification in relation to the calibration standards. The chromatographic parameters for the metabolites analyzed are summarized in Table 3. Statistical analysis was conducted to compare metabolite levels between fentanyl-dependent rats and controls.

### 2.8. Data Analysis

To quantify the microbiota load in fecal samples, a standard series of dilutions of a standard sample was essential for each run of the real-time PCR reaction. The cycle at which the sample fluorescence crossed a preset threshold was marked as the Cq and referred to as the standard curve. Samples were loaded in duplicate wells, and the mean value was utilized for data analysis. Raw LC–MS data were processed using vendor software (Skyline) for peak integration, alignment, and normalization. Metabolite concentrations were expressed as absolute quantities (μM/mg tissue) based on standard curves. Correlation analyses were performed to assess relationships between microbial abundance and metabolic/inflammatory markers, visualized through heatmaps using Spearman or Pearson correlation coefficients. Statistical analyses were carried out using GraphPad Prism (v.6) and SPSS version 26 software. Results are expressed as mean ± SD, with statistical significance set at a *p*-value less than 0.05.

## 3. Results

### 3.1. Fentanyl Treatment Leads to Modulation of the Gut Microbiota at the Species Level

**Figure 1:**
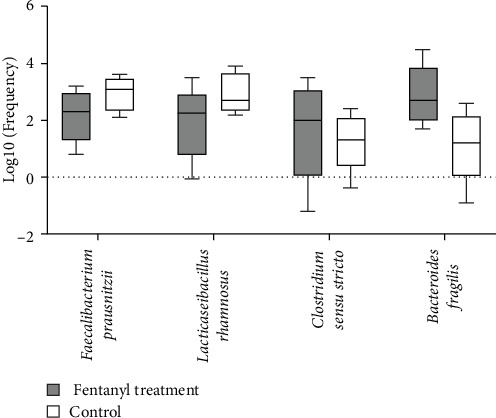
Comparison of abundance of bacteria between fentanyl treated and control groups.

To ascertain the relationship between changes in microbiota and Fentanyl treatment at the species level, an investigation was carried out involving the profiling of selected species-specific 16SrRNA genes (such as *L. rhamnosus*, *F. prausnitzii*, *B. fragilis*, and *C. sensu stricto*) in the gut microbiota through quantitative real-time PCR analysis (as illustrated in Figure 1; Table 4). The findings indicate a proliferation of *C. sensu stricto* species in the group treated with fentanyl (*p*=0.69), which showed a tendency toward increase. Furthermore, a marked rise in the presence of *B. fragilis* was noted in the fentanyl-treated group (*p*=0.04), indicating a significant increase in this bacterial species. Additionally, a reduction in the abundance of *F. prausnitzii* (*p*=0.149) and *L. rhamnosus* (*p*=0.14) was observed in the fentanyl-treated group, with both showing a tendency toward reduction.

### 3.2. Fentanyl Treatment Leads to Intestinal Inflammation

**Figure 2:**
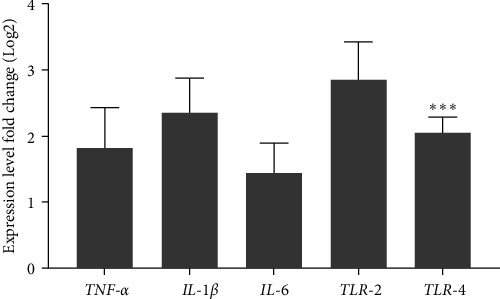
Expression levels of *TNF-α*, *IL-1β*, *IL-6*, *TLR-2*, and *TLR-4* by RT-PCR assays from rats challenged by fentanyl abuse. The obtained data for each experimental group is reported as the mean ± SD (*n* = 10). The distribution of the individual data in each group is also seen on each bar. *⁣*^*∗∗∗*^: *p*  < 0.001.

The intestinal mucosa of rats was examined for the expression levels of *IL-1β*, *TNF-α*, *IL-6*, *TLR-2*, and *TLR-4* using RT-PCR analysis (Figure 2). Compared to the saline control group, rats treated with Fentanyl had higher circulating levels of proinflammatory cytokines and Toll-like receptors (*IL-1β*, *p*=0.01; *TNF-α*, *p*=0.083; *IL-6*, *p*=0.17; and *TLR-2*, *p*=0.005). Notably, a significant disparity in the expression of *TLR-4* was detected between the Fentanyl-treated and control groups (*p*=0.001).

### 3.3. Fentanyl Treatment Leads to Metabolic Pathway Alterations

**Figure 3:**
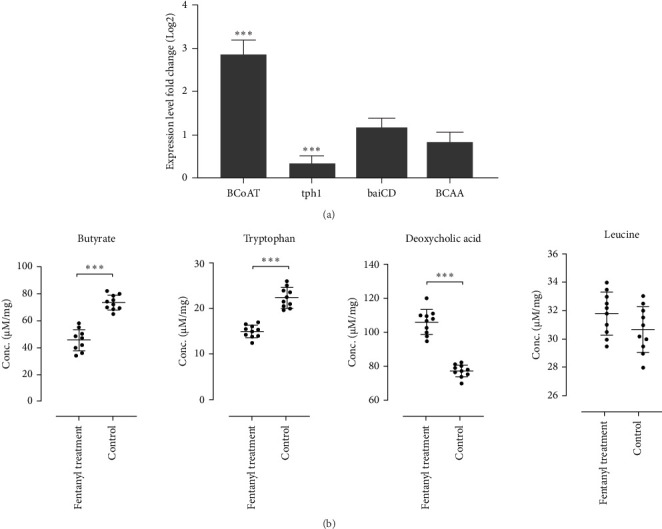
Analysis of metabolites in fentanyl-dependent rats and control groups. (A) Expression levels of *BCoAT*, *tph1*, *baiCD*, and *BCAA* by RT-PCR assays from rats challenged by fentanyl abuse. (B) Concentrations (µM) of butyrate, tryptophan, deoxycholic acid, and leucine measured using LC–MS. The obtained data for each experimental group is reported as the mean ± SD (*n* = 10). *⁣*^*∗∗∗*^: *p*  < 0.001.

The analysis of metabolic pathways revealed significant changes in gene expression related to the inflammatory response (Figure 3A). The expression levels of *BCoAT* were significantly increased in fentanyl-treated rats compared to controls (*p*  < 0.001). In contrast, the expression of *tph1* decreased significantly in fentanyl-treated rats when compared to controls (*p*  < 0.001). The expression of *baiCD* showed a slight increase in fentanyl-treated rats compared to controls (*p*=0.64), although this change was not statistically significant. Similarly, *BCAA* expression demonstrated a slight decrease in fentanyl-treated rats compared to controls (*p*=0.03), with no significant differences observed.

On the other hands, the levels of butyrate, tryptophan, deoxycholic acid, and leucine were quantified in intestinal tissue samples from fentanyl-dependent rats and control groups using LC–MS analysis (Figure 3B). Analysis revealed a significant decrease in butyrate levels in the intestines of fentanyl-dependent rats (45.49 ± 7.82 µM) compared to controls (73.52 ± 5.4 µM; *p*  < 0.001). This reduction suggests impaired SCFA production, potentially affecting gut barrier integrity. Tryptophan concentrations were also significantly lower in fentanyl-dependent rats (14.96 ± 1.3 µM) compared to the control group (22.38 ± 2.1 µM, *p*  < 0.001). This finding indicates a possible disruption in serotonin synthesis, which could influence mood regulation and pain perception in these animals. Deoxycholic acid levels showed a significant increase in fentanyl-dependent rats (106.1 ± 7.3 µM) compared to controls (77.35 ± 3.5 µM, *p*  < 0.001). This elevation may suggest altered bile acid metabolism, potentially contributing to inflammatory responses in the gut. Leucine levels were comparable between groups, with fentanyl-dependent rats showing a mean concentration of 31.8 ± 1.5 µM versus 30.67 ± 1.6 µM in controls (*p*=0.15). This suggests that BCAA metabolism may remain relatively unaffected by fentanyl dependance. Statistical comparisons were confirmed significant differences in butyrate, tryptophan, and deoxycholic acid levels between the fentanyl-dependent and control groups. No significant difference was observed for leucine levels. Overall, these results indicate notable alterations in gut metabolite profiles associated with fentanyl dependance, highlighting the potential impact on gut health and metabolic functions.

### 3.4. Correlations Among Microbiota, Inflammatory, and Metabolic Markers in Fentanyl-Treated Subjects

**Figure 4:**
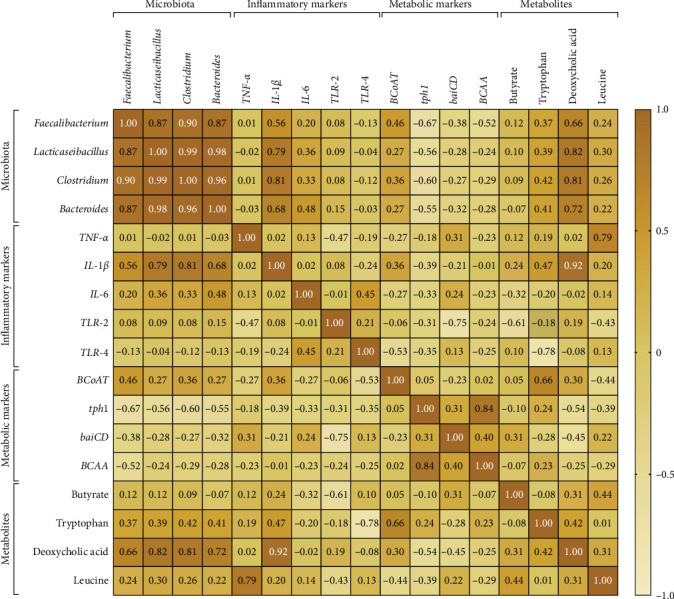
Heatmap representation of Spearman/Pearson correlation analyses between specific bacterial abundance and inflammatory/metabolic markers in the fentanyl dependance rat model. Heatmap depicting Spearman/Pearson correlation coefficients between significantly altered microbial taxa (*Faecalibacterium prausnitzii*, *Lacticaseibacillus rhamnosus*, *Clostridium sensu stricto*, and *Bacteroides fragilis*) and inflammatory markers (*TNF-α*, *IL-1β*, *IL-6*, *TLR-2*, and *TLR-4*) metabolic markers (*BCoAT*, *tph1*, *baiCD*, and *BCAA*) and metabolites (butyrate, tryptophan, deoxycholic acid, and leucine). Tendency to value 1 indicates positive correlations, to value −1 indicates negative correlations, and 0 denotes no significant association (*⁣*^*∗*^*p*  < 0.05, *⁣*^*∗∗*^*p*  < 0.01, *⁣*^*∗∗∗*^*p*  < 0.001).

The Spearman/Pearson correlation analysis identified significant associations among microbiota genera, inflammatory markers, and metabolic genes in the fentanyl-treated group. The correlation matrix can be found in Figure 4, while the statistical significance of the correlations is detailed in Table 5. Strong positive correlations were observed among specific beneficial bacteria. *F. prausnitzii* showed high correlations with *L. rhamnosus* (*r* = 0.87; *p*  < 0.001) and *C. sensu stricto* (*r* = 0.90; *p*=0.0004), suggesting interdependence among these genera. *B. fragilis* similarly correlated with *L. rhamnosus* (*r* = 0.98; *p*  < 0.001) and *C. sensu stricto* (*r* = 0.96; *p*  < 0.001), indicating that shifts in one genus may affect the others in response to fentanyl treatment. In contrast, *F. prausnitzii* displayed weaker correlations with inflammatory markers, such as *TNF-α* (*r* = 0.01; *p*=0.5) and *IL-6* (*r* = 0.20; *p*=0.2). However, *L. rhamnosus* had moderate positive correlations with *IL-1β* (*r* = 0.79; *p*=0.004) and *IL-6* (*r* = 0.36; *p*=0.1), suggesting that this genus may be involved in the inflammatory response. Notably, *C. sensu stricto* and *B. fragilis* also exhibited positive correlations with *IL-1β* (*r* = 0.81; *p*=0.003; *r* = 0.68; *p*=0.01, respectively), indicating that these bacteria could contribute to inflammation in the fentanyl-treated context. *F. prausnitzii* correlated negatively with *tph1* (*r* = −0.67; *p*=0.01) and BCAA (*r* = −0.52; *p*=0.05), while positively correlating with *BCoAT* expression (*r* = 0.46; *p*=0.05). This suggests that higher *F. prausnitzii* levels may be linked to increased butyrate production, improving gut health, while a decrease in *tph1* and *BCAA* implies reduced serotonin synthesis and impaired amino acid metabolism. Similar negative correlations with *tph1* and *BCAA* were noted for *L. rhamnosus* (*r* = −0.56, *p*=0.04; *r* = −0.24, *p*=0.2), *C. sensu stricto* (*r* = −0.60, *p*=0.03; *r* = −0.29, *p*=0.2), and *B. fragilis* (*r* = −0.55, *p*=0.05; *r* = −0.28, *p*=0.2), highlighting their potential roles in metabolic disruptions associated with fentanyl dependance. Significantly, butyrate levels positively correlated with *F. prausnitzii* (*r* = 0.12; *p*=0.3) and *L. rhamnosus* (*r* = 0.10; *p*=0.3), indicating that these beneficial bacteria facilitate butyrate production, which is crucial for maintaining gut barrier integrity. Additionally, tryptophan levels showed a positive correlation with deoxycholic acid (*r* = 0.42; *p*=0.1) and a moderate correlation with *BCAA* (*r* = 0.23; *p*=0.2), suggesting that microbiota alterations may affect key metabolites critical for gut function.

Strong correlations among inflammatory markers were observed, with *IL-1β* positively correlating with *IL-6* (*r* = 0.02; *p*=0.4) and *TNF-α* (*r* = 0.02; *p*=0.4). *TLR-4* was highly correlated with both *IL-6* (*r* = 0.45; *p*=0.09), indicating a pronounced inflammatory response triggered by fentanyl, likely contributing to gut dysfunction. Moreover, *IL-1β* was positively correlated with *BCoAT* (*r* = 0.36, *p*=0.1), while negatively correlating with *tph1* (*r* = −0.39, *p*=0.1), signifying that heightened inflammation may limit butyrate production, while disrupting serotonin metabolism. *TNF-α* also correlated positively with Leucine (*r* = 0.79; *p*=0.004), indicating an association between inflammation and BCAA metabolism. Conversely, Tryptophan negatively correlated with *TLR-4* (*r* = −0.78; *p*=0.005), suggesting that reduced tryptophan levels may exacerbate inflammatory processes. Overall, these results underscore complex interactions among gut microbiota, inflammation, and metabolism in fentanyl-treated subjects, highlighting the importance of these dynamics in understanding the gut-brain axis' role in opioid dependance and gut health.

## 4. Discussion

The current study highlights the intricate interplay between fentanyl treatment, gut microbiota modulation, intestinal inflammation, and metabolic pathway alterations in a fentanyl dependance rat model. The analysis revealed that fentanyl treatment leads to significant changes in gut microbiota composition, particularly at the species level. The observed proliferation of *C. sensu stricto* (*p*=0.69) and *B. fragilis* (*p*=0.04) in the fentanyl-treated group suggests a shift toward potentially pro-inflammatory microbial community, possibly as a compensatory mechanism to counteract opioid-induced dysbiosis. Although the increase in *C. sensu stricto* was noted, the higher *p*-value indicates that the change is not statistically significant. This suggests that while there may be a trend toward higher levels of this bacterium in fentanyl-treated rats, the evidence is not strong enough to draw definitive conclusions. *B. fragilis* is recognized for its beneficial roles in gut health and immune function; its marked increase may indicate an adaptive response to the inflammatory state induced by fentanyl. Conversely, the reductions in *F. prausnitzii* (*p*=0.14) and *L. rhamnosu*s (*p*=0.14) could highlight a detrimental impact of fentanyl on these beneficial bacteria, potentially compromising gut integrity and contributing to metabolic dysfunction. The tendency towards reduced abundance in these probiotics may lead to decreased production of SCFAs, such as butyrate, known for its protective role in maintaining gut barrier integrity and modulating inflammation.

The current study also demonstrated elevated levels of inflammatory cytokines, including *IL-1β* (*p*=0.01), *TNF-α* (*p*=0.083), and *IL-6* (*p*=0.17), coupled with increased expression of toll-like receptors, particularly *TLR-2* (*p*=0.005) and *TLR-4* (*p*=0.001). The notable elevation of *TLR-4* indicates a robust inflammatory response triggered by fentanyl, which may contribute to altered gut permeability and function. This finding aligns with existing literature that associates opioid use with intestinal inflammation, which in turn compromises overall gastrointestinal health and fosters a vicious cycle of dysbiosis and inflammation. Furthermore, the metabolic pathway analyses revealed significant disruptions linked to fentanyl treatment. Specifically, the increased expression of *BCoAT* (*p*  < 0.001), critical for butyrate synthesis, alongside a significant decrease in *tph1* (*p*  < 0.001), which is essential for serotonin production, suggests that opioid exposure may hinder the creation of beneficial metabolites while promoting metabolic dysregulation. Although *baiCD* showed a slight increase (*p*=0.64), this change was not significant, and *BCAA* expression demonstrated a slight decrease (*p*=0.03). Notably, the decrease in butyrate and tryptophan levels (*p*  < 0.001) illustrates the impact of fentanyl on SCFA production and neurotransmitter synthesis, emphasizing potential adverse consequences for gut barrier function, mood regulation, pain perception, and overall health. The increased levels of deoxycholic acid (*p*  < 0.001) further signify altered bile acid metabolism, which may contribute to inflammatory processes in the gut.

Correlational analysis further elucidated the complex relationships among microbiota, inflammatory markers, and metabolic variables in the fentanyl-treated group. The negative correlations of *F. prausnitzii* with *tph1* and *BCAA* suggest that higher levels of *F. prausnitzii* may be associated with lower serotonin production and BCCAs, indicating a potential metabolic influence on gut health and inflammation. The positive correlation with *BCoAT* implies a potential role in bile acid metabolism; however, despite increased *BCoAT* expression, actual butyrate levels were reduced, indicating potential substrate availability issues or impaired metabolic pathways for butyrate production that could lead to gut health issues. Additionally, the decrease in *tph1* expression correlates with reduced tryptophan levels, highlighting disruption in serotonin synthesis that may impact mood and pain perception. The observed correlation between inflammatory markers and microbial species reinforces the idea that disruptions in gut microbiota may exacerbate inflammatory responses. For instance, the significant correlations between *L. rhamnosus*, *C. sensu stricto*, and *B. fragilis* with *IL-1β* suggest that these species may contribute to inflammation in the context of fentanyl treatment. These findings are consistent with previous studies in both animals and humans that have reported similar changes following opioid use [13, 29–31].

The changes observed in the composition of gut microbiota, specifically the increase in *C. sensu stricto* and *B. fragilis*, as well as the decrease in *F. prausnitzii* and *L. rhamnosus*, could contribute to the disruption of immune responses and inflammatory processes in the gut [32]. While an increase in *C. sensu stricto* has been linked to elevated inflammatory reactions due to toxin production, previous research indicates that this genus can enhance the proliferation of other harmful bacteria, intensifying the inflammatory response [25, 33]. Previous research has indicated a rise in *B. fragilis* levels in individuals engaging in opioid abuse [26]. The increase in this specific bacterium levels observed in this study might reflect its capacity to activate inflammatory pathways by producing toxins that stimulate immune responses through the NF-*κ*B pathway [34]. This interplay hints at a compromised immune response in opioid users, potentially resulting in chronic inflammation. Both *F. prausnitzii* and *L. rhamnosus* are important for enhancing helper T cell levels and reducing inflammation [35]. *F. prausnitzii* is a butyrate-producing bacterium that helps induce inflammation [36], with butyrate being crucial for gut health due to its protective anti-inflammatory effects [37]. Individuals who abuse opioids exhibit lower levels of *F. prausnitzii*, which correlates with increased body inflammation and contributes to chronic inflammation associated with fentanyl abuse, leading to various health complications [38]. Similarly, research indicates that opioid abuse results in decreased levels of *L. rhamnosus*, which is known for its anti-inflammatory properties and ability to regulate immune responses [39]. Prior research has also indicated a decrease in *L. rhamnosus* species with opioid abuse [10]. The reduction in *L. rhamnosus* abundance may amplify inflammatory responses and compromise intestinal barrier integrity. This dysbiosis underscores the significance of maintaining a healthy gut microbiome in individuals with substance use disorders to mitigate the adverse effects of fentanyl abuse. Moreover, the underlying mechanisms of fentanyl's disruption of gut microbiota and inflammation are complex and multifaceted. Fentanyl's interaction with the MOP receptor in both the ENS and immune cells likely alters gut motility and secretion [40], facilitating dysbiosis where harmful bacteria (*e.g*., *C. sensu stricto* and *B. fragilis*) thrive while beneficial ones (*e.g*., *F. prausnitzii* and *L. rhamnosus*) decline [24, 41]. Fentanyl may also damage the gut barrier, increasing intestinal permeability—commonly referred to as “leaky gut”—which allows bacterial toxins to enter the bloodstream and activate pro-inflammatory signaling pathways like TLR-4/NF-*κ*B, subsequently elevating pro-inflammatory cytokines, such as IL-6, IL-1β, and TNF-α [42, 43]. Additionally, fentanyl dysregulates the hypothalamic-pituitary-adrenal (HPA) axis, elevating cortisol and further disrupting gut microbiota [15]. Understanding these processes opens avenues for potential treatment strategies. Targeting specific gut bacteria, as well as inflammatory and metabolic markers, could serve as biomarkers for opioid-related gut disorders. For instance, utilizing probiotics, such as *F. prausnitzii* and *L. rhamnosus* might help restore microbial balance [44], while dietary strategies to enhance butyrate production could mitigate the inflammatory effects of fentanyl-induced dysbiosis [45]. Drugs targeting opioid receptors or TLR signaling to mitigate gut inflammation in those affected by opioid abuse [46]. Overall, findings of this study contribute valuable insights into the diagnosis and treatment of gastrointestinal disorders and systemic inflammation and metabolic pathwayes related to opioid use. The observed changes in gut microbiota may serve as significant biomarkers for identifying individuals at risk of developing opioid-related complications [24]. Furthermore, the increase in inflammatory/metabolic markers can provide additional diagnostic and prognostic value, equipping clinicians with the tools to monitor inflammation and make more informed treatment decisions [47].

While the study revealed significant alterations in certain gut microbiota following fentanyl abuse, some changes, including those involving *C. sensu stricto* and *L. rhamnosus*, did not reach statistical significance. These findings may reflect natural variations in gut microbiota or limitations related to sample size and study design. Importantly, the lack of functional prediction analyses due to our reliance on absolute real-time PCR limits a complete understanding of the biological implications of these microbial changes. Nonetheless, the trends observed suggest biological significance and warrant further investigation. Future research employing larger sample sizes, longer follow-up periods, and advanced techniques like metagenomics will be pivotal for confirming these results and elucidating the underlying mechanisms. Longitudinal studies could help establish causal relationships between fentanyl use, gut microbiota changes, and inflammation, while randomized controlled trials could evaluate the efficacy and safety of potential interventions, such as probiotics and prebiotics in individuals with opioid use disorder. The integration of multiomics approaches, combining metagenomics with metabolomics and proteomics, may provide a more comprehensive understanding of how opioid-induced dysbiosis contributes to inflammation, potentially leading to more personalized treatment strategies [31]. A deeper comprehension of the precise mechanisms linking gut microbiota alterations to intestinal inflammation in opioid users could yield important insights into the development of opioid-related gastrointestinal disorders and inform novel approaches to managing opioid addiction [15]. Additional research is needed to fully uncover how gut microbiota composition influences inflammatory responses in the context of opioid use, with the ultimate goal of developing targeted interventions to alleviate opioid-induced gastrointestinal complications.

In essence, this study enhances our understanding of the interactions between fentanyl use, gut microbiota changes, and intestinal inflammation, emphasizing the necessity of addressing gastrointestinal health in opioid-dependent individuals. The findings suggest that disturbances in these systems may contribute to systemic inflammation in this population. Future research should focus on investigating fentanyl's direct effects on specific gut bacterial communities and inflammatory pathways, as well as exploring potential strategies to modulate gut microbiota and immune function in the context of opioid addiction. By clarifying the mechanisms underlying opioid-induced gut dysfunction, researchers may develop more precise approaches to prevent or mitigate inflammation and associated gastrointestinal complications in chronic opioid users. This comprehensive perspective could ultimately lead to improved health outcomes and quality of life for individuals struggling with opioid addiction, bridging the gap between scientific discovery and clinical application.

## 5. Conclusion

In conclusion, current research highlights the complex relationship between fentanyl consumption, gut microbiota species, intestinal inflammation and metabolic pathways. Significant alterations in specific gut bacteria were observed, particularly an increase in *B. fragilis* and a trend toward elevated levels of *C. sensu stricto*, both of which may correlate with heightened inflammatory responses in the intestines. The findings indicate significant elevations in pro-inflammatory cytokines and toll-like receptors, notably *TLR-4*, linking fentanyl exposure to intestinal inflammation. Disruptions in metabolic pathways, alongside decreased production of beneficial metabolites like butyrate and tryptophan, further emphasize the metabolic consequences of fentanyl dependance. Moreover, correlation analyses reveal intricate relationships between specific microbiota species and inflammatory and metabolic markers, suggesting these interactions are crucial to the inflammatory processes associated with opioid use. Understanding these relationships is essential for exploring therapeutic strategies to restore gut balance in individuals with opioid dependency. By elucidating the role of gut microbiota and their metabolic products in opioid-induced inflammation, it may be identified new treatment targets to improve recovery outcomes. Overall, these findings stress the necessity of a comprehensive approach to address gut health and its implications in opioid addiction, aiming for enhanced health outcomes for affected individuals.

## Figures and Tables

**Table 1:** 16S rRNA gene universal primer sequences used for quantitative RT-PCR reaction.

Bacteria	Forward	Reverse	Amplicon size (bp)
*Faecalibacterium* *prausnitzii*	GGAGGAAGAAGGTCTTCGG	AATTCCGCCTACCTCTGCACT	248

*Lacticaseibacillus rhamnosus*	AGCAGTAGGGAATCTTCCA	CACCGCTACACATGGAG	341

*Bacteroides* *fragilis*	AGAGTTTGATCCTGGCTCAG	GTCATCGTGCACACAGAATTGCTG	117

*Clostridium* *sensu stricto*	TTGAGCGATTTACTTCGGTAAAGA	CCATCCTGTACTGGCTCACCT	157

**Table 2:** Primer sequences for inflammatory and metabolic markers analyzed by RT-PCR.

Gene group	Gene	Forward	Reverse	Amplicon size (bp)
Cytokine genes	*TNF*-α	AAATGGGCTCCCTCTCATCAGTTC	TCTGCTTGGTGGTTTGCTACGAC	271
	*IL-1*β	CACCTCTCAAGCAGAGCACAG	GGGTTCCATGGTGAAGTCAAC	276
	*IL-6*	TCCTACCCCAACTTCCAATGCTC	TTGGATGGTCTTGGTCCTTAGCC	168

Toll-like receptors	*TLR-2*	TCTGAGTTCCGTGACATAGG	AGATGTAACGCAACAGATTC	169
	*TLR-4*	GTGAGCATTGATGAGTTCAG	CATCTAATGATTGATAAGGATT	170

Metabolic pathways-related genes	*BCoAT*	GACATGGTGCTGGTGGTTTC	TCCGCACCTTCACATTCTTG	150
	*tph1*	GCTGAACAAACTCTACCCAACCCAC	TCGGCACAGTCCAAACTCCACA	169
	*baiCD*	GGCGATGGTATTGACGGTGT	TCCAGGTCCGATGTTCAGGT	120
	*BCAA*	GATGGTGGTACCGCTGAAGA	TCGTCGTTCAGCTTCAGGTT	120

Housekeeping gene	*gapdh*	ATCAACGACCCCTTCATTGACC	CCAGTAGACTCCACGACATACTA	204

**Table 3:** Summary of chromatographic parameters for selected metabolites, including retention times (RT), precursor and quantifier m/z values, optimized collision energies (CE), calibration curve ranges, and internal standards used in the analysis.

Metabolite	RT (mins)	Precursor [M + H]+ (m/z)	Quantifier m/z	Qualifier m/z	Optimized CE (eV)	Calibration curve range (µg/mL)	Internal standard
Butyrate	3.5	86	86	74	10	10–100	Sodium butyrate-d5
Tryptophan	5.0	204	204	188	20	5–250	Tryptophan-d5
Deoxycholic acid	7.2	392	392	374	15	10–100	Cholic acid
Leucine	6.0	132	132	116	25	5–50	Leucine-d3

**Table 4:** Comparison of abundance of bacteria between fentanyl treated and control groups*⁣*^*∗*^.

Bacteria	Fentanyl treated		Control		*p*-Value*⁣*^*∗∗∗*^
	Mean	SD	Mean	SD	
*Faecalibacterium prausnitzii* CFU*⁣*^*∗∗*^ (× 10^3^)	2.16	0.91	2.94	0.59	0.149417
*Lacticaseibacillus rhamnosus* CFU (× 10^3^)	1.868	1.29	2.94	0.69	0.140503
*Clostridium sensu stricto* CFU (× 10^3^)	1.64	1.78	1.26	1.04	0.691414
*Bacteroides fragilis* CFU (× 10^3^)	2.88	1.05	1.12	1.28	0.045903

*⁣* ^*∗*^The data are presented as mean ± SD.

*⁣* ^*∗∗*^Colony-forming unit.

*⁣* ^*∗∗∗*^ *p*-Value < 0.05 was considered statistically significant.

**Table 5:** Statistical significance of correlations between gut microbiota and inflammatory/metabolic markers in fentanyl-dependent rats.

Variables	*Faecalibacterium* *prausnitzii*	*Lacticaseibacillus rhamnosus*	*Clostridium* *sensu stricto*	*Bacteroides* *fragilis*	*TNF-α*	*IL-1β*	*IL-6*	*TLR-2*	*TLR-4*	*BCoAT*	*tph1*	*baiCD*	*BCAA*	Butyrate	Tryptophan	Deoxycholic acid	Leucine
*Faecalibacterium* *prausnitzii*	—	<0.001 *⁣*^*∗∗∗*^	0.0004 *⁣*^*∗∗∗*^	<0.01 *⁣*^*∗∗*^	0.5 –	0.04 *⁣*^*∗*^	0.2 –	0.4 –	0.3 –	0.09 –	0.01 *⁣*^*∗*^	0.1 –	0.06 –	0.3 –	0.1 –	0.02 *⁣*^*∗*^	0.2 –

*Lacticaseibacillus rhamnosus*	<0.001 *⁣*^*∗∗∗*^	—	<0.001 *⁣*^*∗∗∗*^	<0.001 *⁣*^*∗∗∗*^	0.4 –	0.004 *⁣*^*∗∗*^	0.1 –	0.4 –	0.4 –	0.2 –	0.04 *⁣*^*∗*^	0.2 –	0.2 –	0.3 –	0.1 –	0.002 *⁣*^*∗∗*^	0.2 –

*Clostridium* *sensu stricto*	0.0004 *⁣*^*∗∗∗*^	<0.001 *⁣*^*∗∗∗*^	—	<0.001 *⁣*^*∗∗∗*^	0.5 –	0.003 *⁣*^*∗∗*^	0.1 –	0.4 –	0.3 –	0.1 –	0.03 *⁣*^*∗*^	0.2 –	0.2 –	0.4 –	0.1 –	0.003 *⁣*^*∗∗*^	0.2 –

*Bacteroides* *fragilis*	<0.01 *⁣*^*∗∗*^	<0.001 *⁣*^*∗∗∗*^	<0.001 *⁣*^*∗∗∗*^	—	0.4 –	0.01 *⁣*^*∗*^	0.08 –	0.3 –	0.4 –	0.2 –	0.05 –	0.1 –	0.2 –	0.4 –	0.1 –	0.01 *⁣*^*∗*^	0.2 –

*TNF-α*	0.5 –	0.4 –	0.5 –	0.4 –	—	0.4 –	0.3 –	0.08 –	0.3 –	0.2 –	0.3 –	0.1 –	0.2 –	0.3 –	0.3 –	0.4 –	0.004 *⁣*^*∗∗*^

*IL-1β*	0.04 *⁣*^*∗*^	0.004 *⁣*^*∗∗*^	0.003 *⁣*^*∗∗*^	0.01 *⁣*^*∗*^	0.4 –	—	0.4 –	0.4 –	0.2 –	0.1 –	0.1 –	0.2 –	0.5 –	0.2 –	0.08 –	< 0.001 *⁣*^*∗∗∗*^	0.2 –

*IL-6*	0.2 –	0.1 –	0.1 –	0.08 –	0.3 –	0.4 –	—	0.5 –	0.09 –	0.2 –	0.17 –	0.2 –	0.2 –	0.1 –	0.2 –	0.4 –	0.3 –

*TLR-2*	0.4 –	0.4 –	0.4 –	0.3 –	0.08 –	0.4 –	0.5 –	—	0.2 –	0.4 –	0.1 –	0.007 *⁣*^*∗∗*^	0.2 –	0.03 *⁣*^*∗∗*^	0.3 –	0.3 –	0.1 –

*TLR-4*	0.3 –	0.4 –	0.3 –	0.4 –	0.3 –	0.2 –	0.09 –	0.2 –	—	0.05 *⁣*^*∗*^	0.1 –	0.3 –	0.2 –	0.3 –	0.005 *⁣*^*∗∗*^	0.4 –	0.3 –

*BCoAT*	0.05 *⁣*^*∗*^	0.2 –	0.1 –	0.2 –	0.2 –	0.1 –	0.2 –	0.4 –	0.05 *⁣*^*∗*^	—	0.4 –	0.2 –	0.4 –	0.4 –	0.02 *⁣*^*∗*^	0.1 –	0.1 –

*tph1*	0.01 *⁣*^*∗*^	0.04 *⁣*^*∗*^	0.03 *⁣*^*∗*^	0.05 –	0.3 –	0.1 –	0.1 –	0.1 –	0.1 –	0.4 –	—	0.1 –	<0.001 *⁣*^*∗∗∗*^	0.3 –	0.2 –	0.05 *⁣*^*∗*^	0.1 –

*baiCD*	0.1 –	0.2 –	0.2 –	0.17 –	0.1 –	0.2 –	0.2 –	0.007 *⁣*^*∗∗*^	0.3 –	0.2 –	0.1 –	—	0.1 –	0.1 –	0.2 –	0.09 –	0.2 –

*BCAA*	0.05 *⁣*^*∗*^	0.2 –	0.2 –	0.2 –	0.2 –	0.5 –	0.2 –	0.2 –	0.2 –	0.4 –	<0.001 *⁣*^*∗∗∗*^	0.1 –	—	0.4 –	0.2 –	0.2 –	0.2 –

Butyrate	0.3 –	0.3 –	0.4 –	0.4 –	0.3 –	0.2 –	0.1 –	0.03 *⁣*^*∗*^	0.3 –	0.4 –	0.3 –	0.1 –	0.4 –	—	0.4 –	0.1 –	0.1 –

Tryptophan	0.1 –	0.1 –	0.1 –	0.1 –	0.3 –	0.08 –	0.2 –	0.3 –	0.005 *⁣*^*∗∗*^	0.02 –	0.2 –	0.2 –	0.2 –	0.4 –	—	0.1 –	0.5 –

Deoxycholic acid	0.02 *⁣*^*∗*^	0.003 *⁣*^*∗∗*^	0.004 *⁣*^*∗∗*^	0.01 *⁣*^*∗*^	0.4 –	<0.001 *⁣*^*∗∗∗*^	0.4 –	0.3 –	0.4 –	0.1 –	0.05 *⁣*^*∗*^	0.09 –	0.2 –	0.1 –	0.1 –	—	0.1 –

Leucine	0.2 –	0.2 –	0.2 –	0.2 –	0.004 *⁣*^*∗∗*^	0.2 –	0.3 –	0.1 –	0.3 –	0.1 –	0.1 –	0.2 –	0.2 –	0.1 –	0.5 –	0.1 –	—

*Note: p*-Values derived from Spearman/Pearson correlation analyses are shown, with significance thresholds set at *⁣*^*∗*^*p*  < 0.05, *⁣*^*∗∗*^*p*  < 0.01, and *⁣*^*∗∗∗*^*p*  < 0.001. NS = not significant (*p*  ≥ 0.05).

## Data Availability

The data sharing is not applicable to this article as no new data were created or analyzed in this study.
